# Robotic assisted versus laparoscopic surgery for deep endometriosis: a meta-analysis of current evidence

**DOI:** 10.1007/s11701-024-01954-2

**Published:** 2024-05-16

**Authors:** Matteo Pavone, Alessandro Baroni, Federica Campolo, Marta Goglia, Diego Raimondo, Antonella Carcagnì, Cherif Akladios, Jacques Marescaux, Francesco Fanfani, Giovanni Scambia, Manuel Maria Ianieri

**Affiliations:** 1 UOC Ginecologia Oncologica, Dipartimento di Scienze per la salute della Donna e del Bambino e di Sanità Pubblica, Fondazione Policlinico Universitario A. Gemelli, IRCCS, Largo Agostino Gemelli 8, 00168 Rome, Italy; 2 Institute of Image-Guided Surgery, IHU Strasbourg, Strasbourg, France; 3 Research Institute against Digestive Cancer, IRCAD, Strasbourg, France; 4 Department of Medical and Surgical Sciences and Translational Medicine, Faculty of Medicine and Psychology, Sapienza University of Rome, Rome, Italy; 5 Division of Gynecology and Human Reproduction Physiopathology, Department of Medical and Surgical Sciences (DIMEC), IRCCS, Sant’Orsola-Malpighi Hospital, University of Bologna, Bologna, Italy; 6 Facility of Epidemiology and Biostatistics - Gemelli Generator, Fondazione Policlinico Universitario “A. Gemelli” IRCCS, Largo A. Gemelli, 8, 00168 Rome, Italy; 7 Department of Gynecologic Surgery, University Hospitals of Strasbourg, Strasbourg, France; 8 Università Cattolica del Sacro Cuore, Rome, Italy; 9 Gynecology and Breast Care Center, Mater Olbia Hospital, Olbia, Italy

**Keywords:** Robotic assisted surgery, Endometriosis, Minimally Invasive surgery, Image-guided surgery, RAS, Robotic platforms

## Abstract

Endometriosis is a benign inflammatory onco-mimetic disease affecting 10–15% of women in the world. When it is refractory to medical treatments, surgery may be required. Usually, laparoscopy is the preferred approach, but robotic surgery has gained popularity in the last 15 years. This study aims to evaluate the safety and efficacy of robotic-assisted laparoscopic surgery (RAS) versus conventional laparoscopic surgery (LPS) in the treatment of endometriosis. This study adheres to PRISMA guidelines and is registered with PROSPERO. Studies reporting perioperative data comparing RAS and LPS surgery in patients with endometriosis querying PubMed, Google Scholar and ClinicalTrials.gov were included in the analysis. The Quality Assessment of Diagnostic Accuracy Studies 2 tool (QUADAS-2) was used for the quality assessment of the selected articles. Fourteen studies were identified, including 2709 patients with endometriosis stage I-IV for the meta-analysis. There were no significant differences between RAS and LPS in terms of intraoperative and postoperative complications, conversion rate and estimated blood loss. However, patients in the RAS group have a longer operative time (*p* < 0.0001) and longer hospital stay (*p* = 0.020) than those in the laparoscopic group. Robotic surgery is not inferior to laparoscopy in patients with endometriosis in terms of surgical outcomes; however, RAS requires longer operative times and longer hospital stay. The benefits of robotic surgery should be sought in the easiest potential integration of robotic platforms with new technologies. Prospective studies comparing laparoscopy to the new robotic systems are desirable for greater robustness of scientific evidence.

**Supplementary Information:**

The online version contains supplementary material available at 10.1007/s11701-024-01954-2.

## Introduction

Endometriosis, is an “onco-mimetic” inflammatory disease influenced by estrogen, that impacts the 10–15% of women in their reproductive age [1]. It primarily presents in the pelvic region, manifesting as superficial peritoneal implants, ovarian endometriomas, or “deep” lesions extending beyond the peritoneal surface (> 5 mm), commonly found in areas like the uterosacral ligaments, rectouterine pouch, vagina, bowel, bladder, and ureters. Symptoms vary based on the location and may include dysmenorrhea, chronic pelvic pain, dyspareunia, infertility, and urinary and intestinal function impairment [2]. Surgical excision of lesions is considered recommended if hormonal treatments prove insufficient to manage the symptoms [3, 4], in case of bowel or ureteral stricture or in selective case of infertility [4]. Minimally invasive surgical (MIS) approaches have become predominant in the surgical management of the disease, with laparoscopy as a standard of care [4]. Despite its advantages, conventional laparoscopy has limitations such as 2-dimensional visualization, ergonomic limits, and a restricted range of instruments. Over the past decade, the viability, efficacy, and safety of robotic-assisted surgery (RAS) in addressing deep endometriosis has been reported, demonstrating its non-inferiority to laparoscopy [5]. Robotic systems offer enhanced depth perception, wrist articulation, and dexterity, particularly beneficial for complex cases or challenging anatomical locations like diaphragmatic endometriosis or sites involving the sacral plexus or ischial nerves [6, 7]. The use of robotic articulated instruments, equipped with clutching mechanisms that exceed the range of motion of the human wrist (> 360°), facilitates access to these areas. However, the lack of tactile feedback and the associated high costs of installation and maintenance present obstacles to the widespread adoption of RAS [8]. Despite established benefits in various surgical domains, the superiority of RAS over traditional laparoscopy in treating endometriosis remains unknown [9]. The aim of this meta-analysis is to compare the effectiveness and safety of these approaches in the surgical management of endometriosis.

## Methods

The review was conducted according to Preferred Reporting Items for Systematic Reviews and Meta-Analyses (PRISMA) guidelines [10]. Before data extraction, the review was registered with the International Prospective Register of Systematic Reviews (PROSPERO, Registration N° CRD CRD42023495700).

## Eligibility criteria

According to the PICO [10] schema were selected articles focused on comparison between robotic assisted and laparoscopic surgery in deep endometriosis regarding at least one of the following parameters: (i) intraoperative complications (ii) postoperative complications (iii) operative time (iv) conversion rate (v) estimated blood loss (vi) hospital stay. Articles not reporting comparisons between the two surgical approaches were excluded. Only full-text studies were considered eligible for inclusion. Abstracts, reviews, meta-analyses, letters, case reports and editorials were excluded (Table 1).

**Table 1:** Study Characteristics

Author	Year	Study type	Group	Sample size (*n*)	Age (mean, SD)	BMI	rASRM(12) stage
Nezhat et al. [14]	2010	Retrospective	LPS RAS	38 40	33 (18–46) 35 (22–49)	23 (18–31) 24 (19–37)	I–IV
Dulemba et al. [15]	2013	Retrospective	LPS RAS	100 180	29.2 ± 9.2 32.6 ± 9.7	26.8 ± 11.9 27.9 ± 7.7	I–IV
Nezhat et al. [20]	2014	Retrospective	LPS RAS	86 32	40 ± 4.5 42.5 ± 2.2	24.53 ± 1.2 27.36 ± 2.5	III–IV
Nezhat et al. [19]	2015	Retrospective	LPS RAS	273 147	31 ± 5.7 30 ± 2.5	23 ± 2.5 23 ± 3.2	III–IV
Magrina et al. [21]	2015	Retrospective	LPS RAS	162 331	38.3 ± 10.7 40 ± 10.1	25.5 ± 5.7 26.1 ± 5.9	III–IV
Soto et al. [5]	2017	Prospective	LPS RAS	38 35	34.5 ± 8.5 34.3 ± 7.2	24.8 ± 5.9 26.1 ± 5.2	I–IV
Le Gac et al. [22]	2020	Prospective	LPS RAS	25 23	37 ± 8 36 ± 7	25 ± 4 25 ± 3	III–IV
Hiltunen et al. [16]	2021	Retrospective	LPS RAS	76 18	NA NA	26 (19–39) 24 (18–38)	I–IV
Raimondo et al. [23]	2021	Retrospective	LPS RAS	22 22	36 ± 5 38 ± 7	22.5 (21–24) 24.5 (21–27)	III–IV
Ferrier et al. [17]	2022	Retrospective	LPS RAS	61 61	35 ± 7 36 ± 7	26 ± 8 25 ± 5	I–IV
Legendri et al. [18]	2022	Retrospective	LPS RAS	28 26	34 (27.5–37.5) 36.5(29.7–43.5)	23 (21–29) 23 (20.5–27.5)	IV
Crestani et al. [26]	2023	Retrospective	LPS RAS	73 89	NA NA	26 (19–39) 24 (18–38)	III–IV
Volodarsky Perel et al. [24]	2023	Retrospective	LPS RAS	451 97	37.9 (31.7–44.1 37.3 (30.5–44.1)	22.6 (20.3–25.6) 23.2 (21.3–26.9)	III–IV
Verrelli et al. [25]	2023	Retrospective	LPS RAS	104 71	38.4 (31.5–45.3) 37.3 (31.4–43.2)	23.6 (19.5–27.7) 23.8 (18.8–28.8)	III–IV

## Search strategy

The studies included for analysis were obtained querying the PubMed database, Google Scholar and ClinicalTrial.gov between September and November 2023, filtered only by English language and publication year (1980–2023). The search strategy is reported in the supplementary material (Online Supplementary A).

## Study selection

Rayyan software (Qatar Computing Research Institute, HBKU, Doha, Qatar) [11] was used independently by two authors (MP and AB) to screen titles and abstracts for eligibility. Manual searches were performed on pertinent resources and online links, and references of selected articles were examined. Duplicate entries were eliminated during the title/abstract review. For all relevant studies, the complete text was reviewed by both authors independently. Discordant assessments were resolved by consultation of a third author (MG).

## Data collection

Data collection included: author, publication year, country, sample size, age, BMI, rASRM [12], stage previous surgery, intra- and postoperative reported data. We will provide our data for independent analysis by a selected team or for additional data analysis or for the reproducibility of this study in other centers if such is requested.

## Assessment of risk of bias

The risk of bias was assessed independently by two reviewers (MP and AB) using the Quality Assessment of Diagnostic Accuracy Studies 2 (QUADAS-2) tool [13]. The risk of bias was assessed for the following domains: patient selection, index test, reference standard, and flow and timing. Discordant assessments were resolved by consultation of a third author (MG).

## Analysis and data synthesis

Statistical analyses were performed using R statistical software (version 4.2.1) meta e metaplus statistical package of the software R was used. Risk Ratios (RRs) alongside their 95% confidence intervals (CIs) for intra-, postoperative complications and conversion rates data were extracted from the studies or calculated. To continue variables (operative time (min) OT, estimated blood loss (EBL) and hospitalization stay) SMD were calculated. A random-effects model was used to take the source of heterogeneity related to the clinical setting into account. To assess heterogeneity between studies, the Cochrane’s *Q* test and *I*^2^ index were used. *p* values of < 0.05 were considered as valid for heterogeneity tests. Pooled estimations and the related 95% CIs were evaluated using forest plots. A funnel plot was depicted for the detection of publication bias.

## Results

### Study selection and characteristics

**Fig. 1:**
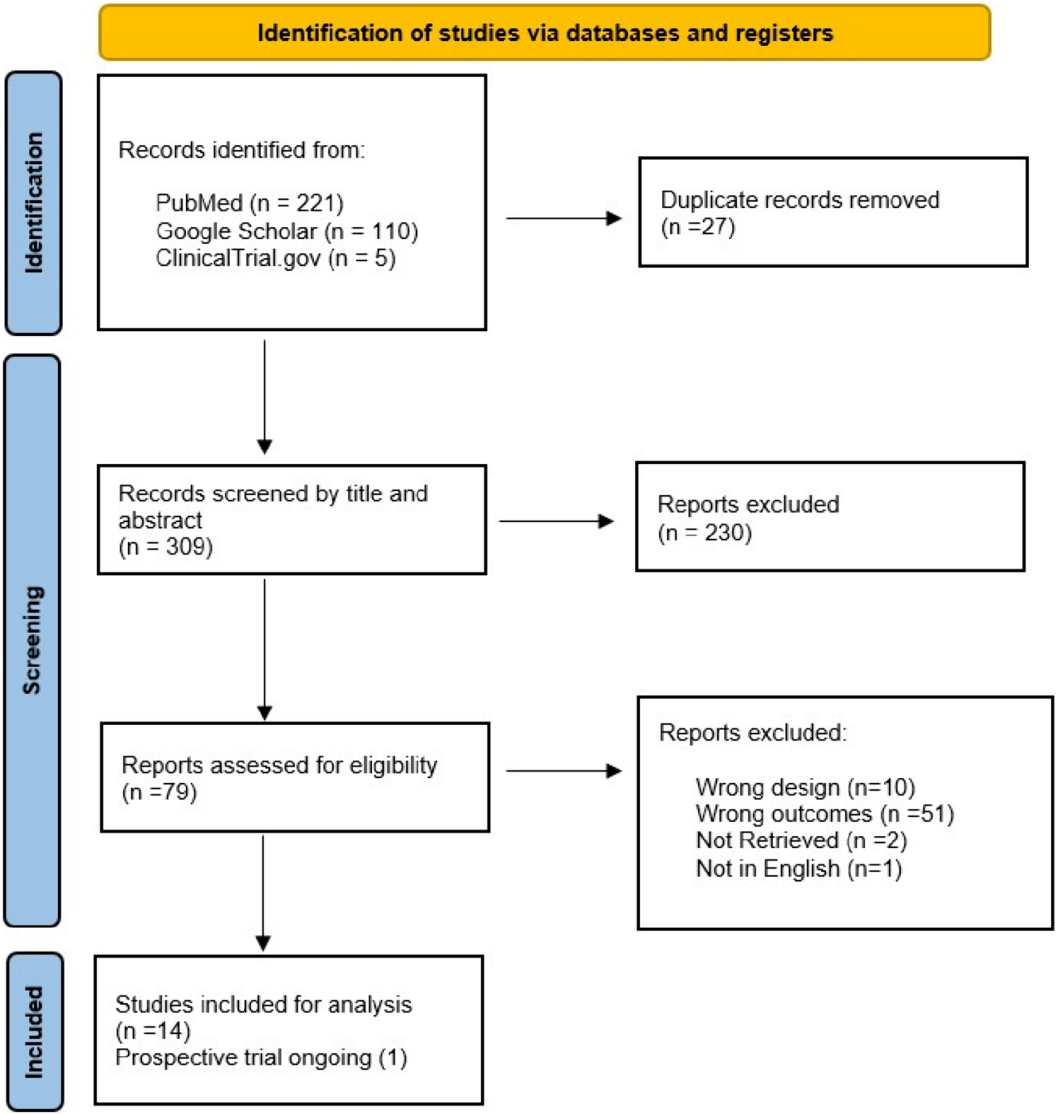
PRISMA Flow diagram for study selection

The initial search identified 340 articles. After removing duplicates and title/abstract screening, 79 manuscripts were assessed for eligibility. Of these, were excluded as they addressed a different outcome (51) or a different design (10) or were inaccessible (2) or in a language different than English (1). A list of excluded articles is provided in Online Supplementary B. Consequently, fourteen studies were included for data synthesis (Online Supplementary C) and one prospective trial was identified. The PRISMA flow diagram shows the complete review process from the original search to the final selection (Fig. 1). The Fourteen studies selected for the meta-analysis covered a total of 2709 patients. Of these twelve (85.7%) are retrospective and 2 prospective (14.3%).

### Risk of bias of included studies

The quality assessment of the included studies is presented in Online Supplementary D. Most studies were at low risk of bias regarding patient selection, index test, and reference standard domains (8, 61,5%).

Five articles had an unclear risk of bias in the patient’s selection as they reported data on patients without differentiating the rASRM stage [5, 14–17] while one focused only on stage IV [18]. One was at an unclear risk of bias and applicability in patient selection due to the exclusion of women undergoing bladder ureteral or bowel resection [19].

### Meta-analysis

#### Intra- and postoperative complications

**Fig. 2:**
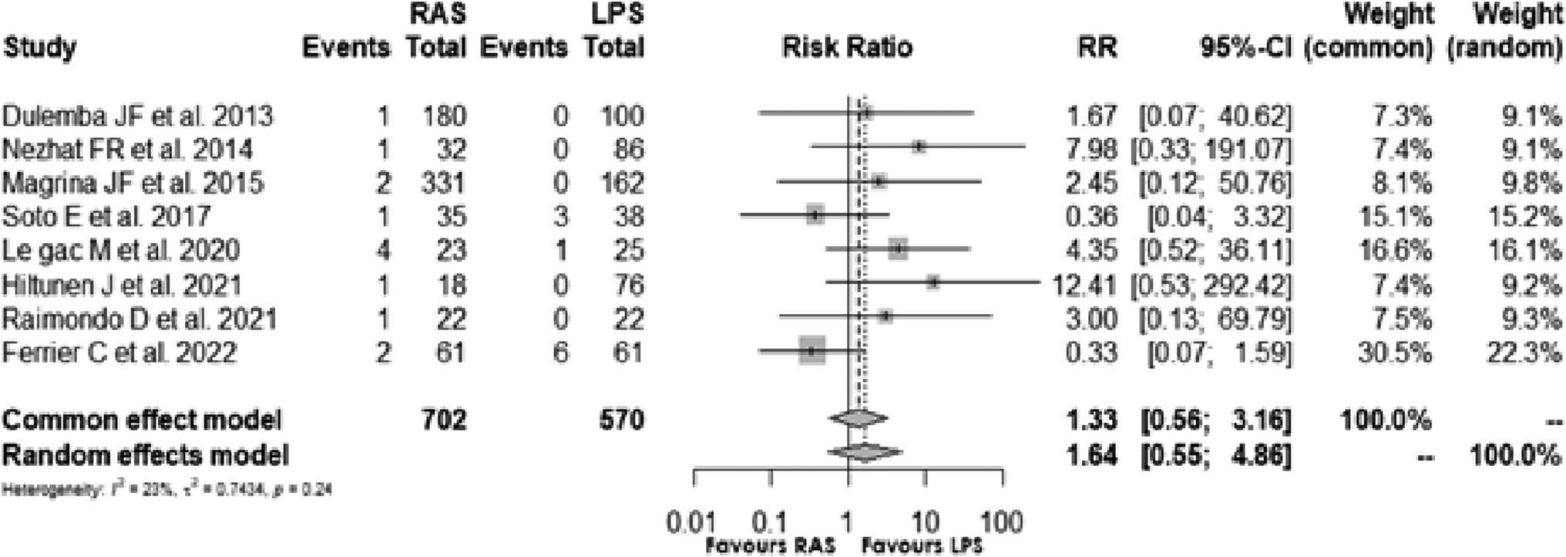
Forest plot for intraoperative complications comparing RAS with LPS

Eight [5, 15–17, 20–23] studies assessed the intra-operative complications of RAS and LPS surgical procedures: the Risk Ratio (RR) of 1.638, 95% CI [0.552; 4.855] and *p* = 0.373, indicated no significant difference between RAS and LPS. The I2 was 23.3%, and test of heterogeneity suggested low statistical heterogeneity **(**Fig. 2**)**.

**Fig. 3:**
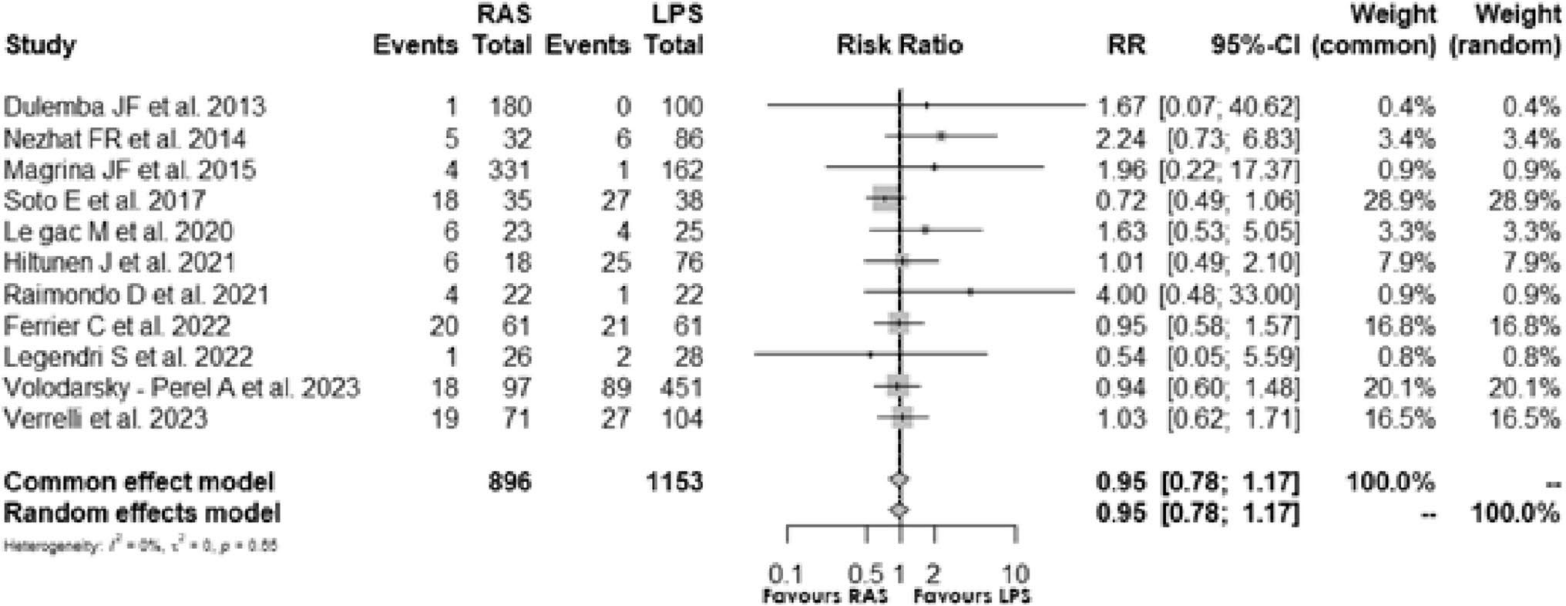
Forest plot for postoperative complications comparing RAS with LPS

Eleven [5, 15–18, 20–25] studies assessed the post-operative complication of RAS and LPS surgical procedures: the Risk Ratio (RR) of 0.952, 95% CI [0.776; 1.169] and *p* = 0.642, indicated no significant difference between RAS and LPS. The I2 was 0.0%, and test of heterogeneity suggested low statistical heterogeneity **(**Fig. 3**)**.

### Conversion rate

**Fig. 4:**
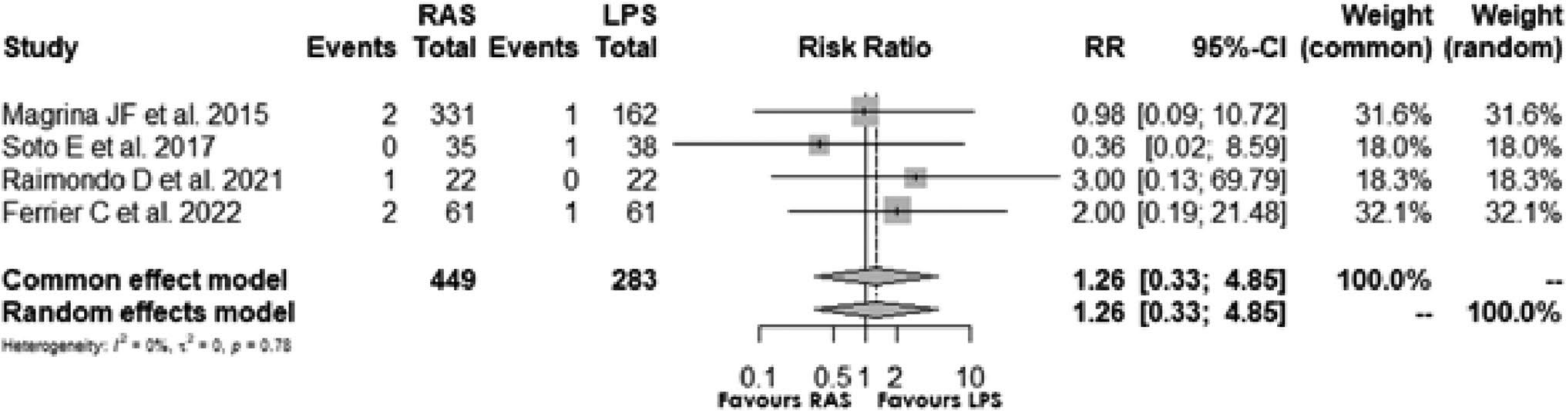
Forest plot for conversion rates comparing RAS with LPS

Four [5, 17, 21, 23] studies assessed the conversion rates of RAS and LPS surgical procedures: the Risk Ratio (RR) of 1.262, 95% CI [0.328; 4.846] and *p* = 0.734, indicated no significant difference between RAS and LPS. The *I*^2^ was 0.0%, and the test of heterogeneity suggested low statistical heterogeneity **(**Fig. 4).

### Operative time

**Fig. 5:**
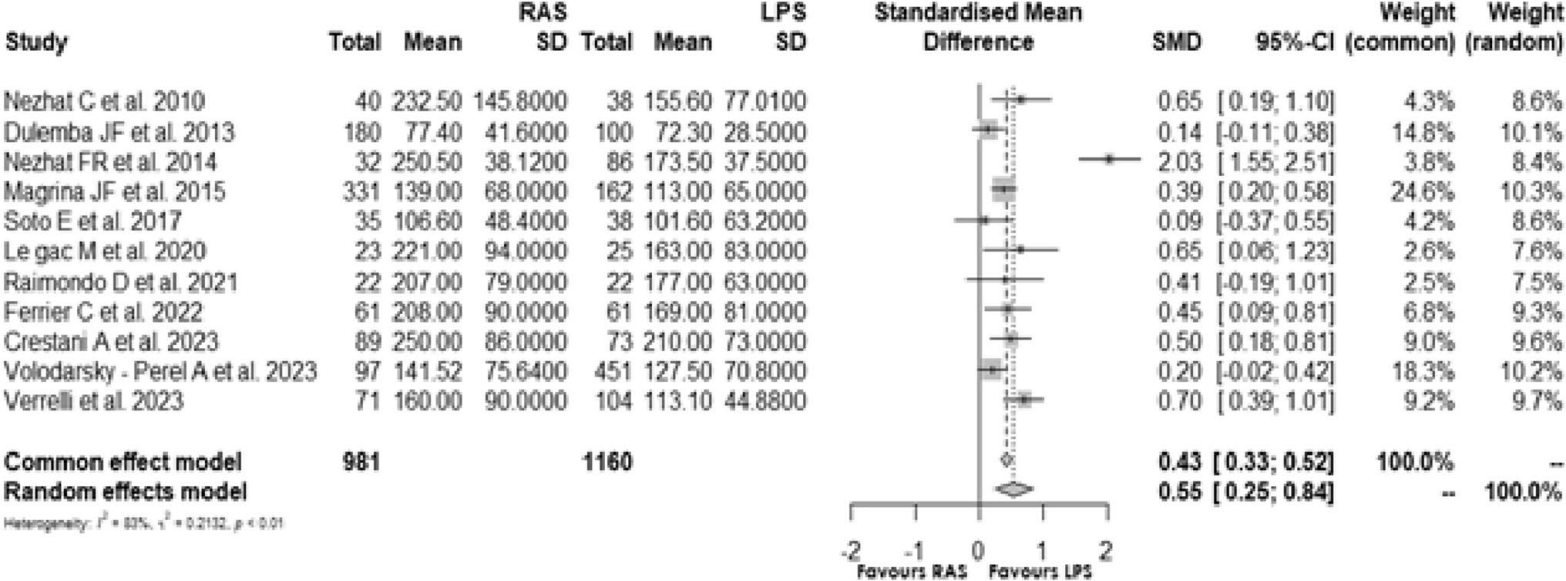
Forest plot for operative time comparing RAS with LPS

Eleven [5, 14, 15, 17, 20–23, 25–27] studies assessed the operative time of the two surgical procedures. The standardisation mean difference (SMD) of 0.54 (min), 95% CI [0.247; 0.842] and *p* < 0.0001, shows that the patients in the RAS group have a longer operative time than those of the laparoscopic group. The *I*^2^ was 83% and the Cochrane’s *Q* test significant results (*p* < 0.0001) suggested high statistical heterogeneity between studies **(**Fig. 5).

### Estimated blood loss

**Fig. 6:**
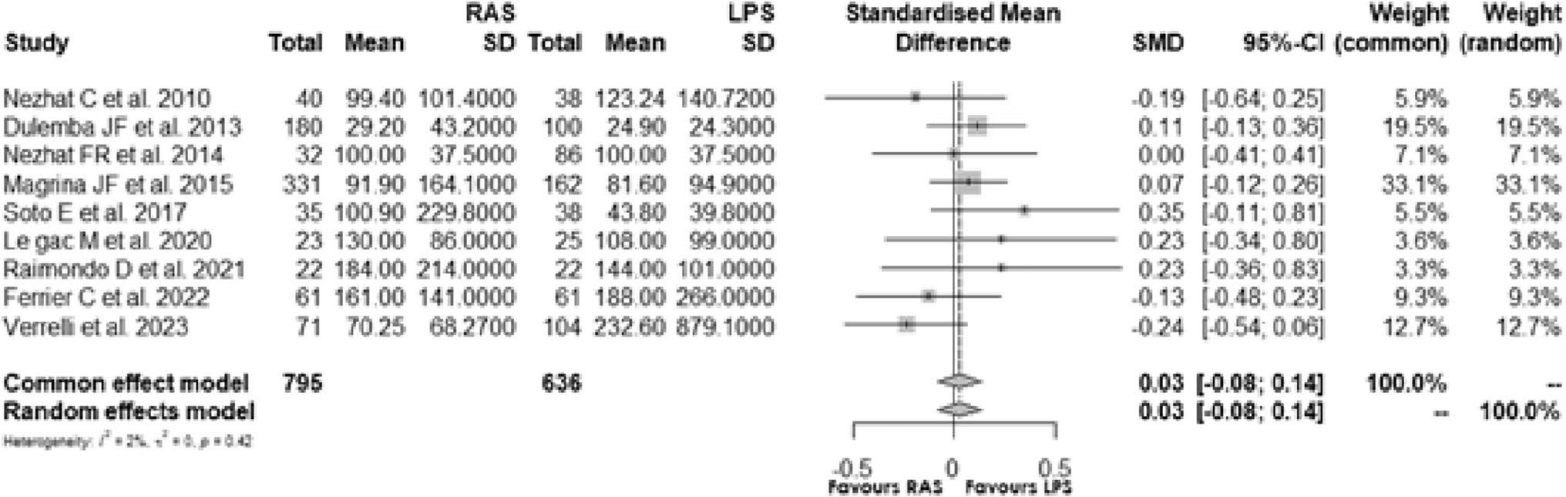
Forest plot for blood loss comparing RAS with LPS

Nine [5, 14, 15, 17, 20–23, 25] studies assessed the estimated blood loss of RAS and LPS surgical procedures: the standardisation mean difference (SMD) of 0.028, 95% CI [− 0.080; 0.136] and *p* = 0.616, indicated no significant difference between RAS and LPS. The *I*^2^ was 1.8%, and the test of heterogeneity suggested low statistical heterogeneity **(**Fig. 6**)**.

### Length of hospital stay

**Fig. 7:**
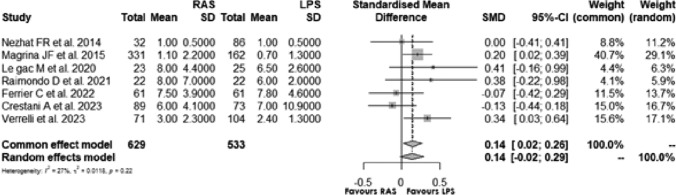
Forest plot for the length of hospitalization comparing RAS with LPS

Seven [17, 20–23, 25, 26] studies assessed hospitalization stay of RAS vs LPS surgical procedures: the standardisation mean difference (SMD) of 0.135, 95% CI [0.022; 0.262] and *p* = 0.020, indicated a significant difference between RAS and LPS. The *I*^2^ was 26.7%, and the test of heterogeneity suggested low statistical heterogeneity **(**Fig. 7**).**

## Discussion

The results of this meta-analysis show the absence of significant differences between the robotic-assisted surgery and the standard laparoscopic approach for endometriosis surgery in terms of intraoperative and postoperative complications, conversion rate and estimated blood loss. However, patients in the RAS group have a longer operative time (*p* < 0.0001) and longer hospital stay (*p* = 0.020) than those in the laparoscopic group.

These results confirm what was previously reported in the metanalysis of Restaino et al. comprising 5 articles on the same topic, with no statistical differences for operative outcomes and a longer OT reported for RAS with a weighted mean difference of 0.54 (*p* < 0.00001) [9]. Therefore, discrepancies are reported in the literature regarding OT in RAS procedures for endometriosis. A longer operating time (MD = 28.09 min, CI 11.59–44.59) and an increased average time of use of the operating room (MD = 51.39 min, CI 15.07–87.72;) is also shown by Csirzó et al. in their recent article [28]. However, Magrina et al. [21] after adjusting their findings for age, blood loss, and number of procedures per patient, showed that RAS approach resulted in 16.2% shorter OT than LPS.

A recent prospective multicentre randomized trial (LAROSE trial) enrolling 73 patients with suspicion of pelvic endometriosis, showed a similar OT between RAS and LPS (mean ± SD, 107 ± 48 min vs. 102 ± 63 min) when adjusted to the stage of disease [5]. According to the latter, the study of Raimondo et al. [23] showed no significant difference between the two groups regarding OT.

Among the factors contributing to the extension of the time required to perform robotic surgery is the docking of the platform. However, these times are directly proportional to the team’s experience and decrease with the learning curve [29]. Regarding the longer hospital stay this could be attributable to a bias in the worst health conditions of patients who are candidates for robotic surgery than for LPS (i.e. obesity) [30].

In addition, after two decades of the Da Vinci^®^ surgical robotic system (Intuitive Surgical, California, USA) as the sole protagonist in the field of RAS, the introduction of new robotic platforms on the marketplace with different features (i.e. open consoles, independent bed-side units) may highlight new evidence. The feasibility of surgical interventions for endometriosis using the new HUGO^™^ RAS (Medtronic, Minneapolis, USA) [31, 32] has already been demonstrated while for other new platforms as the Versius (CMR robotics, UK) system studies are ongoing [33]. Robotic single-site surgery for managing endometriosis was carried out by Huang et al. In 12% of cases, an extra port was introduced to facilitate greater precision of instruments and to address a broader surgical field, particularly in instances involving more complex locations [34]. Despite the growing global adoption of robotic surgery and the increased expertise among surgeons, there is currently insufficient evidence to establish the superiority of robotic surgery over standard laparoscopy in endometriosis surgery. The limited reimbursement for robotic procedures and the extended operative time remains significant concerns, particularly when juxtaposed with the absence of discernible differences in perioperative outcomes. It is important to assess the benefits of the development of robotic surgery beyond the comparison of specific outcomes. As the range of available platforms continues to expand, it becomes imperative to precisely delineate the potential advantages and constraints associated with different systems. The crucial task is not solely to choose the most suitable platform for an individual surgeon, but also to pinpoint the optimal system tailored to the specific requirements of single patients or procedures [35].

The current challenge lies in the training of surgeons and the development of the operating room of the future. In the era of digital surgery, robotic platforms serve as computer interfaces capable of integrating various real-time data analysis modalities. This enables advanced systems to provide augmented surgical vision through augmented reality (AR), improved surgical decisions using artificial intelligence (AI), and enhanced surgical manoeuvres through the advancement of robotic instruments [36]. The incorporation of preoperative planning, utilizing 3D acquisition of radiological images, coupled with the utilization of deep learning (DL) algorithms to analyze surgical phases, forms an ideal toolkit for enhancing robotic surgery [37]. This holistic approach aims to reduce intraoperative complications and optimize surgical outcomes by minimizing surgical discrepancies. The operating room is transitioning into a control center akin to an airport control tower, capable of processing 2D/3D inputs derived from preoperative images, environmental and laparoscopic cameras, and patient physiological signals. It then relays outputs to robotic platforms, offering real-time information on the surgeon’s screen during intraoperative processes, such as remaining operating time or the patient’s clinical situation. Image-guided surgery, particularly intraoperative ultrasound, is gaining prominence in robotic surgery [38, 39]. The integration of drop-in ultrasound probes, easily manipulated by robotic graspers, allows access to challenging anatomical spaces [40]. Intraoperative ultrasound, with images projected onto the surgeon’s screen via platforms like Intuitive Surgical’s TilePro, proves beneficial for achieving surgical radicality in endometriosis [41]. Moreover, robotic systems prove beneficial for educational purposes, providing simulators that can democratize training opportunities, even for non-expert surgeons [42].

In this context, the recent published IDEAL Robotics Colloquium proposes recommendations for evaluation during development, comparative study and clinical monitoring of surgical robots—providing practical guidelines for developers, clinicians, patients and healthcare systems [43].

This paper represents the most recent analysis of the current literature on the comparison of RAS and laparoscopy in patients with endometriosis. The inclusion of 5 papers published in the last 24 months, as well as the methodological accuracy and the assessment of the risk of bias are undoubtedly strengths of the work. However, the retrospective nature of most of the included articles and the adoption in all papers of the Da Vinci platform as the only robotic system analysed represent a limitation of this research. Only one prospective trial was found ongoing (NCT05179109) with the aim to examine whether robot-assisted laparoscopy is superior compared to conventional laparoscopy as regards to patient outcome at 6, 12 and 24 months postoperatively, measured by questionnaires concerning the pain symptoms and disease-related quality-of-life. Future studies, including experience with new robotic platforms and comparisons between these, will be needed to better understand the benefits of RAS over conventional laparoscopy.

## Conclusion

In conclusion, robotic surgery is not inferior to laparoscopy in patients with endometriosis in terms of surgical outcomes; however, RAS require longer operative times and longer hospital stays. The benefits of robotic surgery should be sought in the easiest potential integration of robotic platforms with new technologies. Furthermore, prospective studies comparing laparoscopy to the new robotic systems are desirable for greater robustness of scientific evidence.

## Supplementary Information

Below is the link to the electronic supplementary material.

Supplementary file1 (DOCX 15 KB)

Supplementary file2 (DOCX 19 KB)

Supplementary file3 (DOCX 23 KB)

Supplementary file4 (DOCX 77 KB)

## Data Availability

All data generated or analyzed in this review are included in the manuscript and its figures/tables. Further enquiries can be directed to the corresponding author.
