# Supplementary material for: Robotic assisted versus laparoscopic surgery for deep endometriosis: a meta-analysis of current evidence
Source: J Robot Surg. 2024 May 16;18(1):212. doi: 10.1007/s11701-024-01954-2 (PMC11098866; doi:10.1007/s11701-024-01954-2)
Supplement: Supplementary file 1 — Supplementary file1 (DOCX 15 KB) [file 11701_2024_1954_MOESM1_ESM.docx]

| **SEARCH STRATEGY** | | | |
| --- | --- | --- | --- |
| **Database** | **MeSH terms** | **Search Terms in Database** | **Hits (n)** |
| **MEDLINE**  **PubMed** | 1# Laparoscopy | "laparoscopie"[All Fields] OR "laparoscopy"[MeSH Terms] OR "laparoscopy"[All Fields] OR "laparoscopies"[All Fields] | 137,203 |
|  | 2# Robotic | "robot"[All Fields] OR "robot s"[All Fields] OR "robotically"[All Fields] OR "robotics"[MeSH Terms] OR "robotics"[All Fields] OR "robotic"[All Fields] OR "robotization"[All Fields] OR "robotized"[All Fields] OR "robots"[All Fields] | 94,04 |
|  | 3# Surgery | "surgery"[MeSH Subheading] OR "surgery"[All Fields] OR "surgical procedures, operative"[MeSH Terms] OR ("surgical"[All Fields] AND "procedures"[All Fields] AND "operative"[All Fields]) OR "operative surgical procedures"[All Fields] OR "general surgery"[MeSH Terms] OR ("general"[All Fields] AND "surgery"[All Fields]) OR "general surgery"[All Fields] OR "surgery s"[All Fields] OR "surgerys"[All Fields] OR "surgeries"[All Fields] | 5,732,131 |
|  | 4# endometriosis | "endometriosis"[MeSH Terms] OR "endometriosis"[All Fields] OR "endometrioses"[All Fields] | 34,221 |
|  | 5# | 1# AND 2# AND 3# AND 4# | 221 |
|  | **TOTAL 221 RESULTS** | | |
| **Google Scholar** | 1# Laparoscopy | "laparoscopie"[All Fields] OR "laparoscopy"[MeSH Terms] OR "laparoscopy"[All Fields] OR "laparoscopies"[All Fields] | 87700 |
|  | 2# Robotic | "robot"[All Fields] OR "robot s"[All Fields] OR "robotically"[All Fields] OR "robotics"[MeSH Terms] OR "robotics"[All Fields] OR "robotic"[All Fields] OR "robotization"[All Fields] OR "robotized"[All Fields] OR "robots"[All Fields] | 335000 |
|  | 3# Surgery | "surgery"[MeSH Subheading] OR "surgery"[All Fields] OR "surgical procedures, operative"[MeSH Terms] OR ("surgical"[All Fields] AND "procedures"[All Fields] AND "operative"[All Fields]) OR "operative surgical procedures"[All Fields] OR "general surgery"[MeSH Terms] OR ("general"[All Fields] AND "surgery"[All Fields]) OR "general surgery"[All Fields] OR "surgery s"[All Fields] OR "surgerys"[All Fields] OR "surgeries"[All Fields] | 196000 |
|  | 4# endometriosis | "endometriosis"[MeSH Terms] OR "endometriosis"[All Fields] OR "endometrioses"[All Fields] | 63400 |
|  | 5# | 1# AND 2# AND 3# AND 4# | 110 |
|  | **TOTAL 110 RESULTS** | | |
| **ClinicalTrial.Gov** | Endometriosis AND robotic AND surgery AND laparoscopy | | 5 |
|  | **TOTAL 5 RESULTS** | | |
