# Supplementary material for: Robotic assisted versus laparoscopic surgery for deep endometriosis: a meta-analysis of current evidence
Source: J Robot Surg. 2024 May 16;18(1):212. doi: 10.1007/s11701-024-01954-2 (PMC11098866; doi:10.1007/s11701-024-01954-2)
Supplement: Supplementary file 2 — Supplementary file2 (DOCX 19 KB) [file 11701_2024_1954_MOESM2_ESM.docx]

**Excluded studies**

| **YEAR** | **AUTHORS** | **JOURNAL** | **EXCLUSION-REASONS** | **PMID** |
| --- | --- | --- | --- | --- |
| 2012 | Smorgik N. et al. | Obstetrics and gynecology | Wrong study design | 22914467 |
| 2013 | Vitobello D et al. | The journal of obstetrics and gynaecology research | Wrong study design | 22639980 |
| 2016 | Le Carpentier M et al. | Gynecologie, obstetrique & fertilite | Not in English | 27032760 |
| 2017 | Roman H | Journal of gynecology obstetrics and human reproduction | Wrong study design | 28403973 |
| 2018 | Mosbrucker C et al. | Journal of robotic surgery | Wrong study design | 28255736 |
| 2018 | Moon HS | Journal of laparoendoscopic & advanced surgical techniques | Not retrieved | 29932792 |
| 2020 | Lee HJ et al. | European journal of obstetrics, gynecology, and reproductive biology | Wrong study design | 32339857 |
| 2020 | Vizzielli et al. | Acta obstetricia et gynecologica Scandinavica | Wrong study design | 32274789 |
| 2020 | Gupta N et al. | Gynecology and minimally invasive therapy | Wrong study design | 32676281 |
| 2022 | Grigoriadis G | Journal of minimally invasive gynecology | Not retrieved | 35595229 |
| 2022 | Terho AM et al. | BMJ open | Wrong study design | 35851028 |
| 2022 | Dumont S et al. | The international journal of medical robotics + computer assisted surgery : MRCAS | Wrong study design | 34655461 |
| 2022 | Brunes M et al. | European journal of obstetrics, gynecology, and reproductive biology | Wrong study design | 35299012 |
