# Supplementary material for: Robotic assisted versus laparoscopic surgery for deep endometriosis: a meta-analysis of current evidence
Source: J Robot Surg. 2024 May 16;18(1):212. doi: 10.1007/s11701-024-01954-2 (PMC11098866; doi:10.1007/s11701-024-01954-2)
Supplement: Supplementary file 3 — Supplementary file3 (DOCX 23 KB) [file 11701_2024_1954_MOESM3_ESM.docx]

Supplementary C. Included Study

| **YEAR** | **AUTHOR** | **JOURNAL** | **ENDOMETRIOSIS STAGE** | **STUDY DESIGN** | **SAMPLE SIZE** | **AIM** | **CONCLUSION** |
| --- | --- | --- | --- | --- | --- | --- | --- |
| 2010 | Nezhat C et al. | Fertility and Sterility | I - IV | Retrospective | 78 | Endometriosis treatment encompasses the application of both standard laparoscopy and robot-assisted laparoscopy, enabling a comprehensive comparison of the outcomes associated with each approach. | The outcomes for treating endometriosis are equally favorable with both robot-assisted laparoscopic and standard laparoscopic procedures. However, it's important to note that the robotic approach involves a notably longer duration for both the surgery itself and the administration of anesthesia, along with the use of larger trocars. |
| 2012 | Dulemba JF et al. | Journal of robotic surgery | I - IV | Retrospective | 280 | To assess the practicality of using robot-assisted laparoscopic techniques compared to standard laparoscopy in treating pelvic pain in patients with suspected endometriosis. | This research illustrates the practicality of using robot-assisted surgery to address pelvic pain associated with suspected endometriosis, covering various stages of the disease. The study indicates that the technological enhancements of the robot not only enhance visualization and excision of implanted tissue but also potentially enable less experienced surgeons, who may lack the proficiency needed for traditional laparoscopy, to adopt a minimally invasive approach, even in more intricate cases. |
| 2014 | Nezhat FR et al. | Journal of the Society of Laparoendoscopic Surgeons | III - IV | Retrospective | 108 | The primary parameters evaluated in this study included the scope of the surgery, estimated blood loss, duration of the operating room procedure, intraoperative and postoperative complications, as well as the length of stay. These measures were assessed in both standard laparoscopy and robot-assisted laparoscopy. | Although robotic-assisted laparoscopy tends to have a longer operating room time, it seems to be a secure and minimally invasive method for patients. All other perioperative outcomes, such as intraoperative and postoperative complications, are similar to those observed in patients undergoing conventional laparoscopy. |
| 2015 | Nezhat C et al. | Journal of minimally invasive gynecology | III - IV | Retrospective | 420 | To assess both the safety and effectiveness, this study aims to compare robotic-assisted laparoscopy with conventional laparoscopy in the treatment of advanced-stage endometriosis. | Traditional laparoscopy and robotic-assisted laparoscopy prove to be effective approaches for managing advanced stages of endometriosis. Nevertheless, the utilization of robotic technology may extend the operative duration and could potentially lead to an increased length of hospital stay. |
| 2015 | Magrina JF et al. | Journal of minimally invasive gynecology | III - IV | Retrospective | 493 | The goal of this study is to assess perioperative outcomes and identify factors influencing operating time, hospital length of stay (LOS), and complications in patients undergoing the excision of advanced endometriosis. | The durations of the operations, the amount of blood loss, and the occurrence of complications were comparable between robotics and laparoscopy. Operating time emerged as an independent and noteworthy factor influencing postoperative complications and the duration of hospital stay. |
| 2017 | Soto et al. | Fertility and sterility | I - IV | RCT (NCT01556204) | 73 | The aim of this prospective investigation is to explore the comparative effectiveness of robotic-assisted versus conventional laparoscopy in the treatment of endometriosis, with a primary focus on comparing operative times as the primary outcome. Secondary outcomes include assessing perioperative factors and intermediate-term quality of life outcomes. | There were no differences in perioperative outcomes between robotic and conventional laparoscopy |
| 2020 | Le Gac M et al. | Journal of gynecology obstetrics and human reproduction | III - IV | Prospective | 48 | To evaluate and to compare safety, intra and postoperative complications and short postoperative outcomes of robotic to conventional laparoscopy for endometriosis colorectal resection | Robotic surgery is an adequate alternative to conventional laparoscopy for endometriosis colorectal resection |
| 2021 | Hiltunen J et al. | The Journal of international medical research | Retrospective | I - IV | 94 | The objective of this study is to assess the outcomes of minimally invasive surgery for deep infiltrating endometriosis (DIE) within a single tertiary institution. The specific goal is to compare results following conventional laparoscopic surgery versus robotic-assisted laparoscopic surgery at our institution. Additionally, the study aims to evaluate post-surgery quality of life using a specialized questionnaire. | Robotic-assisted laparoscopy is a feasible method to resect deep infiltrating endometriosis, especially in the rectosigmoid area. |
| 2021 | Raimondo D et al. | Acta obstetricia et gynecologica Scandinavica | Prospective | III - IV | 44 | The objective of this study is to compare robot-assisted laparoscopy with standard laparoscopy in patients affected by rectosigmoid endometriosis. The key parameters under consideration include operative time (OT), overall operative room time, blood loss, length of stay, laparotomic conversion rate, perioperative complications, and endometriosis-related symptoms at the 12-month follow-up. | Robot-assisted laparoscopy yields comparable perioperative outcomes to standard laparoscopy in the surgical treatment of rectosigmoid endometriosis, with the exception of a prolonged operative room time. |
| 2022 | Ferrier C et al. | The international journal of medical robotics + computer assisted surgery | Prospective | I - IV | 122 | To assess the surgical outcomes of robotic surgery in comparison to conventional laparoscopy within a substantial group of women undergoing colorectal surgery for endometriosis. The evaluation will consider various techniques of colorectal resection, including rectal shaving, discoid resection, and segmental resection. | The robotic approach offers advantages, particularly in the context of discoid and segmental resections |
| 2022 | Legendri et al. | Journal of clinical medicine | Retrospective | IV | 95 | We assessed the enhancement in the quality of life utilizing the Endometriosis Health Profile EHP-5, both before and two years after minimally invasive surgery for painful deep infiltrating endometriosis (DIE). Our analysis included subgroups, comparing classic laparoscopy versus robotic laparoscopy, as well as conservative surgery versus total surgery. | The most effective surgical approach, comparing robotic and traditional laparoscopy, remains uncertain and requires further evaluation with a larger sample size and in a prospective manner. |
| 2023 | Crestani et al. | Journal of robotic surgery | I – IV | Retrospective | 162 | To assess and contrast the use of pain relievers after surgery within the hospital duration among patients who underwent colorectal resection for endometriosis, comparing those who had the procedure performed using robotic surgery versus conventional laparoscopy | Opioid consumption was not influenced by either the clinical features or the findings observed during surgery |
| 2023 | Volodarsky - Perel A et al. | Colorectal disease: the official journal of the Association of Coloproctology of Great Britain and Ireland | III - IV | Retrospective | 548 | To assess the occurrence of postoperative complications by comparing the outcomes of robotic-assisted and conventional laparoscopic approaches in the treatment of substantial rectal (ENZIAN C3) endometriotic nodules. | The utilization of the robotic-assisted approach did not demonstrate an elevated risk of major postoperative complications when compared to conventional laparoscopy in the treatment of substantial rectal endometriotic nodules. |
| 2023 | Verrelli et al. | Journal of minimally invasive gynecology | III - IV | Retrospective | 175 | Comparing the post-operative outcomes and overall costs in a consecutive series of patients treated exclusively for severe endometriosis, comparing those who underwent conventional laparoscopy with those who underwent robotic surgeries. | Affirms the viability of utilizing a robotic approach in treating severe endometriosis, despite acknowledging the associated elevated costs linked to the robotic method. |
