# Supplementary material for: Robotic assisted versus laparoscopic surgery for deep endometriosis: a meta-analysis of current evidence
Source: J Robot Surg. 2024 May 16;18(1):212. doi: 10.1007/s11701-024-01954-2 (PMC11098866; doi:10.1007/s11701-024-01954-2)

**QUADAS-2 risk of bias graphical representation.**

| **Year** | **Study** | **RISK OF BIAS** | | | | **APPLICABILITY CONCERNS** | | |
| --- | --- | --- | --- | --- | --- | --- | --- | --- |
|  |  | **PATIENT SELECTION** | **INDEX TEST** | **REFERENCE STANDARD** | **FLOW AND TIMING** | **PATIENT SELECTION** | **INDEX TEST** | **REFERENCE STANDARD** |
| 2010 | Nezhat et al 2010 | ? | ☺ | ☺ | ☺ | ☺ | ☺ | ☺ |
| 2013 | Dulemba et al 2013 | ? | ☺ | ☺ | ☺ | ☺ | ☺ | ☺ |
| 2014 | Nezhat et al 2014 | ☺ | ☺ | ☺ | ☺ | ☺ | ☺ | ☺ |
| 2015 | Nezhat et al 2015 | ☺ | ☺ | ☺ | ☺ | ? | ☺ | ☺ |
| 2015 | Magrina et al 2015 | ☺ | ☺ | ☺ | ☺ | ☺ | ☺ | ☺ |
| 2017 | Soto et al 2017 | ? | ☺ | ☺ | ☺ | ☺ | ☺ | ☺ |
| 2020 | Le Gac et al 2020 | ☺ | ☺ | ☺ | ☺ | ☺ | ☺ | ☺ |
| 2021 | Hiltunen 2021 | ? | ☺ | ☺ | ☺ | ☺ | ☺ | ☺ |
| 2021 | Raimondo et al 2021 | ☺ | ☺ | ☺ | ☺ | ☺ | ☺ | ☺ |
| 2022 | Ferrier et al 2022 | ? | ☺ | ☺ | ☺ | ☺ | ☺ | ☺ |
| 2022 | Legendri et al 2022 | ? | ☺ | ☺ | ☺ | ☺ | ☺ | ☺ |
| 2023 | Crestani et al 2023 | ☺ | ☺ | ☺ | ☺ | ☺ | ☺ | ☺ |
| 2023 | Volodarsky Perel et al 2023 | ☺ | ☺ | ☺ | ☺ | ☺ | ☺ | ☺ |
| 2023 | Verrelli et al 2023 | ☺ | ☺ | ☺ | ☺ | ☺ | ☺ | ☺ |

☺Low Risk ☹High Risk ? Unclear Risk


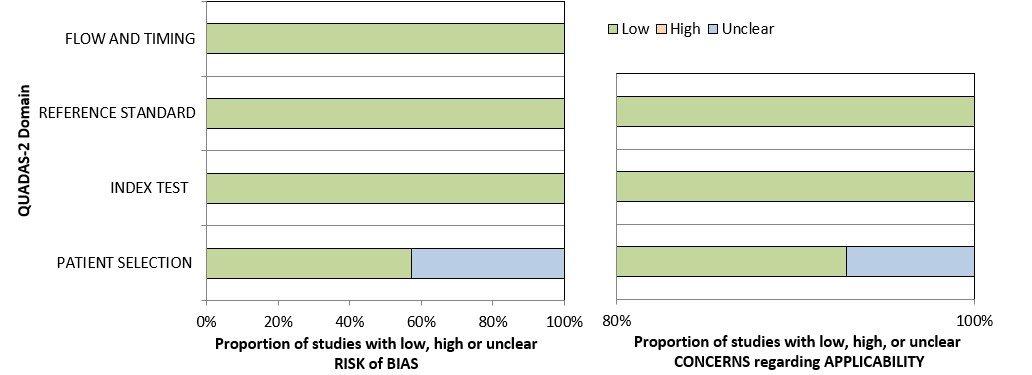

Supplement: Supplementary file 4 — Supplementary file4 (DOCX 77 KB) [file 11701_2024_1954_MOESM4_ESM.docx]
